# Enteroadsorbent Polymethylsiloxane Polyhydrate vs. Probiotic *Lactobacillus reuteri* DSM 17938 in the Treatment of Rotaviral Gastroenteritis in Infants and Toddlers, a Randomized Controlled Trial

**DOI:** 10.3389/fped.2020.553960

**Published:** 2020-12-21

**Authors:** Leo Markovinović, Ivica Knezović, Tihana Kniewald, Lorna Stemberger Marić, Vladimir Trkulja, Goran Tešović

**Affiliations:** ^1^University Hospital for Infectious Diseases “Dr. Fran Mihaljević”, Zagreb, Croatia; ^2^School of Dental Medicine, University of Zagreb, Zagreb, Croatia; ^3^School of Medicine, University of Zagreb, Zagreb, Croatia

**Keywords:** gastroenteritis, children, polymethylsiloxane polyhidrate, *Lactobacillus reuteri* DSM 17938, rotavirus

## Abstract

**Purpose:** The aim of this study was to compare two adjunct therapies in the treatment of childhood rotavirus gastroenteritis (RVGE). We compared the recommended treatment, probiotic *Lactobacillus reuteri* DSM 17938 (BioGaia®), vs. a novel treatment, enterosorbent polymethylsiloxane polyhydrate (Enterosgel®).

**Methods:** This was an open-label, randomized, clinical controlled trial at the University Hospital for Infectious Diseases (UHID) in Zagreb, Croatia. A total of 149 children aged 6–36 months with acute rotaviral gastroenteritis over a period of <48 h, with no significant chronic comorbidity, were randomized to receive the standard therapy with *L. reuteri* DSM 17938 (hereafter *L. reuteri*) or polymethylsiloxane polyhydrate (hereafter PMSPH) therapy, during 5 days. The primary end point was time to recovery in days in both groups. The recovery was defined as absence of fever and vomiting and either the first firm stool, absence of stool for more than 24 h, or return of usual bowel habit.

**Results:** A total of 75 children were randomized into the *L. reuteri* group and 74 were randomized into the PMSPH group; after excluding missing data, the data from 65 children in each group were analyzed. There was no significant difference in the treatment efficacy between the two regimens with an estimated median time of recovery of 6 days in both groups (*p* = 0.754). No significant side effects were observed in either group.

**Conclusion:** Novel enterosorbent PMSPH had a similar efficacy to probiotic *L. reuteri* in the treatment of rotaviral gastroenteritis in preschool children.

**Clinical Trial Registration:** ClinicalTrials.gov Identifier: NCT04116307 [October 3, 2019] (retrospectively registered). https://clinicaltrials.gov/show/NCT04116307.

## The Study

### Background

Rotavirus is the most frequent cause of acute gastroenteritis (AGE) in preschool children in countries without universal infant vaccination against rotavirus (RV) (1, 2). A similar situation can be observed in Croatia since universal vaccination against RV is still not part of the national immunization schedule. Health insurance covers only the vaccination of the population at risk, such as children with heart defects, chronic kidney and liver diseases, metabolic illnesses, and severe brain damage. The reasons for such practice are purely financial. Based on the number of vaccines sold over the counter, we can estimate that only 10% of children have been vaccinated against RV in Croatia in the last few years (3).

The pathogenesis of RV infection consists of direct damage of duodenal enterocytes by the virus, which causes malabsorption, and the toxic effect of viral nonstructural protein 4 (NSP4), which causes increased fluid secretion, damage to tight junctions, and increased intestinal motility (4). Clinical manifestations of RVGE include all three symptoms of AGE—vomiting, fever, and diarrhea, which amplify the loss of water and contribute to the severity of the disease. There is no causative therapy for the treatment of RV infection. The mainstay of treatment is rehydration and antipyretics. Children who do not tolerate oral rehydration therapy (ORT) could be given intravenous solutions of glucose and electrolytes. Several studies have shown that probiotics (including *L. reuteri*) shorten the period of diarrhea in children and reduce the need for hospitalization (5–9). The precise mechanism of action of probiotics is not fully understood. It is known that modification of the gut microbiota can have beneficial effects such as competition with pathogens for food and adherence points, strengthening of the gut epithelial barrier, and modulation of the gut immune system (10). According to the Cochrane Library meta-analysis, probiotic treatment for acute infectious diarrhea shows clear benefits in the shortening of diarrhea duration and reduction of stool frequency (11). Thus, the guidelines of the European Society for Pediatric Gastroenterology, Hepatology and Nutrition (ESPGHAN) and the European Society for Pediatric Infectious Diseases (ESPID) suggest that some probiotics can be considered for the treatment of gastroenteritis. These include the first-line probiotics *Lactobacillus rhamnosus* GG (LGG) and *Saccharomyces boulardii. Lactobacillus reuteri* DSM 17938 and *Lactobacillus acidophilus* LB are also included in the list of recommended strains (12).

Regarding enterosorbent use as an adjunctive therapy for AGE, ESPGHAN/ESPID guidelines only mention diosmectite, a natural mineral clay, to be considered as a therapeutic option (12). Another therapeutic enterosorbent, with proven efficacy in the clinical setting, is the medical device PMSPH (13–16). Its effectiveness is similar to probiotics although the mode of action is different. PMSPH possesses a preferential adsorption capacity for larger molecules, such as bacterial toxins and protein degradation products. It also forms a thin layer over the mucosal surface and thus protects it from various damaging factors (17).

The aim of this randomized non-inferiority study was to compare the efficacy, tolerability, and safety of two adjunct therapies: probiotic *L. reuteri* vs. enterosorbent PMSPH, in the treatment of RVGE in childhood.

## Methods

We carried out a prospective, open-label, randomized, controlled trial from January 1, 2013, until May 31, 2017. The study participants were children treated for RVGE at the outpatient department or hospital ward of the University Hospital for Infectious Diseases (UHID), Zagreb. The inclusion criteria were as follows: age 6–36 months, proven RV infection, duration of illness <48 h before the first visit, and signed written consent by parents or caregivers for participation in the study. The exclusion criteria were as follows: vaccination against RV, previous laboratory-confirmed RV infection in patient's history, and some chronic illnesses or conditions that could influence the course of RV infection (food allergy, malabsorption, liver or pancreatic failure, chronic heart disease, or immune deficiencies). A small number of children (six) started taking probiotics prior to the inclusion into our study for various reasons. If they had taken probiotics different from *L. reuteri* they were excluded from the study. They were also excluded if they had taken *L. reuteri* and were randomized into the PMSPH group. Infants that had taken *L. reuteri* in a small dose for the treatment of previous infant colic were not excluded. Three children were included in the probiotic group, who already started taking *L. reuteri* for infant colic.

All parents/guardians were informed about the survey, both in direct contact with the investigator and by a written letter containing all the details about the study. If they agreed, they signed the informed consent. Children meeting inclusion criteria, whose parents/guardians provided a signed written informed consent, were randomized to *L. reuteri* or PMSPH groups. To achieve the balance between two groups, we used permutated block randomization (random block size 2–6). The randomization list was kept in a sealed envelope by the Department Head Nurse not involved in the patient enrollment or provision of care. Parents/guardians were given a simple daily diary to record their child's symptoms. The diary recorded the following symptoms: frequency of stools, frequency of vomiting, and body temperature of the subject for 8 days from the beginning of the symptoms. Stools were recorded as firm (•), semi-liquid (⊙), and liquid (◦).

The comparative study intervention was the class IIa medical device PMSPH. It was administered orally as recommended in the Instructions for Use, 3 × 10 g dissolved in the same amount of water for the first two days, and 3 × 5 g for the remaining three days. The control treatment was probiotic *L. reuteri*, which was given at a dose of 1.2 × 10^9^ colony-forming units (CFU) per day. All subjects also received parenteral rehydration using isotonic intravenous solutions of 2.5% glucose/half normal saline or pure normal saline. Parallel to intravenous rehydration, or soon after the child's status improved, all children also were given ORT, which is a standard treatment for gastroenteritis of any cause.

The three main symptoms that constitute the clinical presentation of RV infection were measured on a daily basis: fever, vomiting, and liquid stools. At first visit, parents/caregivers were shown how to measure and record symptoms in the diary, and subsequently recorded symptoms themselves. The symptoms were recorded during the 8 days from the beginning of the illness, after which parents/caregivers returned diaries to the site by mail.

The majority of study participants were examined at the UHID pediatric emergency room and admitted to the hospital/day hospital. Some of the patients already hospitalized for other reasons were also included in the study after they acquired nosocomial RV infection. During the hospital stay, hematologic and biochemical tests were performed as well as a rapid stool test for RV and adenovirus (Rota-AdenoGnost test®, BioGnost, Hannover, Germany). For all patients included in the study, we routinely performed complete blood count, blood glucose, urea ± creatinine, Na, K, Cl, aspartate aminotransferase (AST), alanine aminotransferase (ALT), and stool culture. For some patients, we also conducted additional tests such as acid–base balance, urinalysis, abdominal ultrasound, blood, and/or urine culture.

The subjects were also scored according to the Vesikari and Clark Clinical Severity Scoring System, which is used as a standardized methodology primarily across rotavirus vaccine studies (18, 19).

After data collection, the statistical analysis was performed using SAS for Windows 9.4 software licensed at the Zagreb University School of Medicine.

Primary analysis was conducted on a per-protocol analysis. The primary end point was time to recovery in a number of days. The primary end point was analyzed by Kaplan–Meier and Cox proportional hazards modeling. Recovery was defined as absence of fever and vomiting and (a) first firm stool; (b) absence of stool (and other symptoms) for more than 24 h; or (c) returning of the usual bowel habit. It is known that infants can have mushy stools each day during their healthy period.

Study intervention efficacy was tested on three levels: duration of illness, length of hospital stay, and the total number of stools during the defined period (8 days). For the analysis of duration of illness and length of hospital stay, a proportional hazard regression method was used, while for the total number of stools, the Mann–Whitney test was used. The demographic and epidemiological data were analyzed by descriptive statistics methods.

The primary outcome measure of the efficacy was the duration of symptoms measured as time to recovery in days. In a large study comparing several probiotics in 600 children with AGE, the probiotic *L. casei* (*N* = 100) had a median time to recovery of 3.25 days with a range of 2–8 days (20). The sample size calculation for a statistical power of 80% for a non-inferiority test according to given criteria (primary outcome), data of the span, and median of the time to recovery taken from the literature (20) was converted to mean value and standard deviation (3.25 ± 1 day) (21). With an assumption of the same deviation in the test group, for desired power of non-inferiority, it was necessary to have 64 subjects per group (128 in total) (22).

Test treatment was considered non-inferior to control treatment if the upper limit of 97.5% confidence interval for test control did not exceed 0.5 days (i.e., time to recovery with *T* and 97.5% certainty was no longer than 0.5 days with control treatment).

## Results

**Figure 1:**
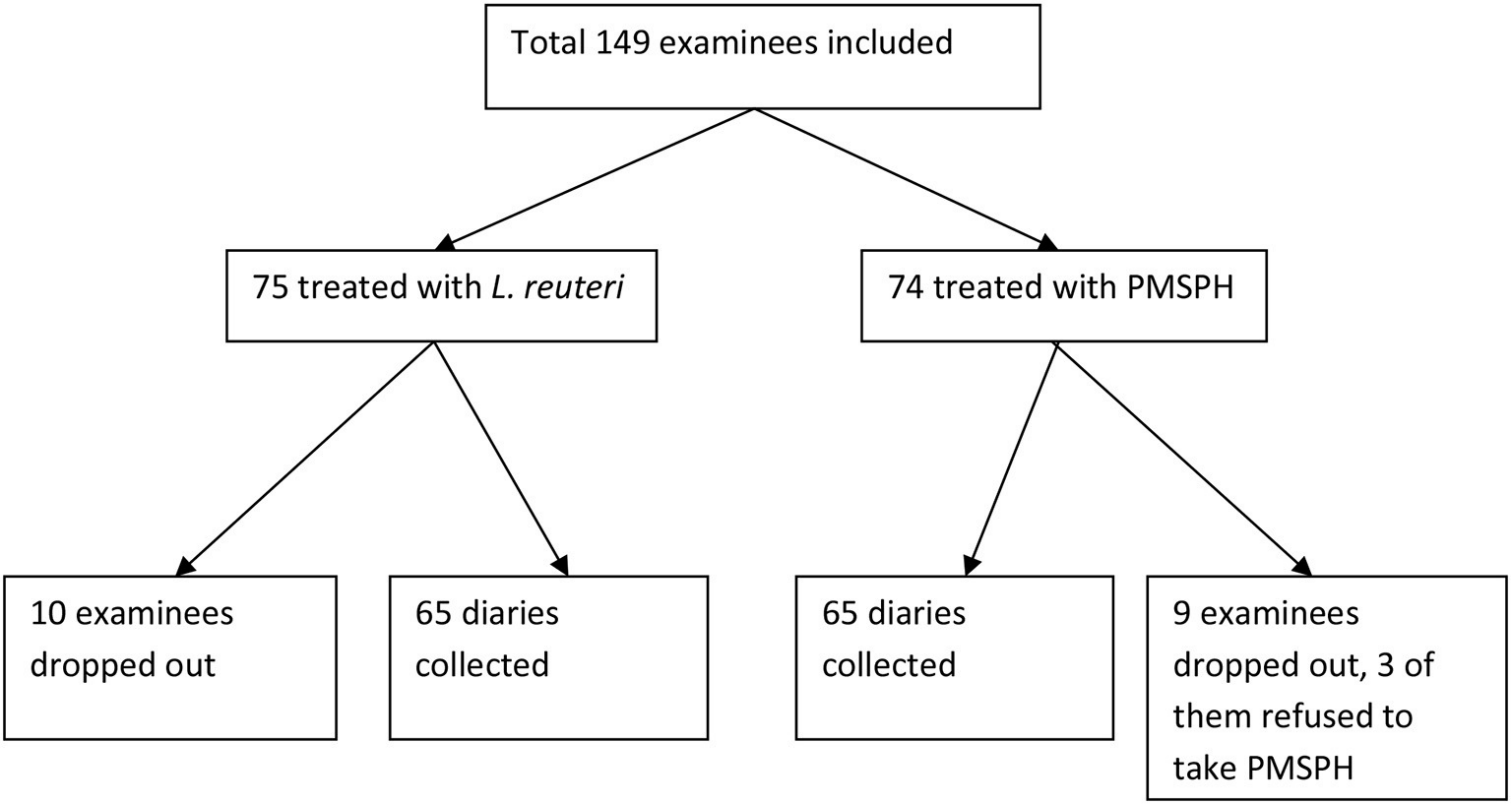
CONSORT flow diagram of the randomized trial of children with rotavirus infection.

The study CONSORT flow diagram (Figure 1) shows the progress of the study participants. Initially, altogether 149 children were enrolled, but only 130 sent back the diary. The data from 130 subjects, 65 from each group, were collected and analyzed.

Children who did not meet the inclusion criteria were discarded by the emergency department doctor, not by the investigator, so the exact number is unknown. The main reason for non-participation was parental refusal to participate and late arrival to the hospital (beyond 48 h after the onset of symptoms). The number of vaccinated children was negligible.

After assessing for eligibility, 149 subjects were randomized to either the *L. reuteri* or the PMSPH group. One hundred thirty subjects completed the study and were analyzed.

The demographic and clinical characteristics in both therapeutic groups were similar except for a somewhat higher frequency of respiratory symptoms or respiratory co-infection in the probiotic group that could influence hospital stay length (Table 1). It is important to emphasize that both groups of children were given ORT alongside test treatments.

**Table 1:** Participant demographics and baseline data summarized by trial arm as analyzed.

**Variable**	**PMSPH (*n* = 65)**	***L. reuteri* (*n* = 65)**
**DEMOGRAPHIC DATA**		
Age (months)	17.4 (6.4–35.8)	15.3 (6.3–35.9)
Sex, male	39 (60.0%)	37 (56.9%)
**CLINICAL CHARACTERISTICS**		
General condition at the beginning of the study		
Good	2 (3.1%)	1 (1.5%)
Fair	47 (72.3%)	49 (75.4%)
Serious	16 (24.6%)	15 (23.1%)
Critical	0	0
Type of rehydration treatment		
Exclusively oral	0	0
Parenteral	65 (100%)	65 (100%)
Vesikari score	18 (11–20)	17 (11–20)
Clark score	15 (9–20)	15 (9–20)
Total number of stools over 8 days	23 (4–86)	25 (6–84)
Respiratory symptoms	11 (16.9%)	17 (26.2%)
Respiratory co-infection	7 (10.8%)	13 (20.9%)
Gastrointestinal co-infection	2 (3.1%)	0 (0)
Hospital-acquired infection	6 (9.2%)	10 (15.4%)
**LABORATORY DATA**		
C-reactive protein (mg/L)	4.0 (0.2–44.8)	4.7 (0.1–41.7)
Total number of leukocytes (× 10^9^/L)	9.5 (3.8–30.6)	10.8 (3.3–27.3)
Percentage of neutrophils	72 (18–92)	67 (10–95)
Blood urea (mmol/L)	5.8 (0.5–10.4)	5.1 (2.4–8.9)
Sodium (mmol/L)	138 (131–144)	138 (132–145)
Potassium (mmol/L)	4.1 (3.2–5.1)	4.3 (3.2–5.2)
Chloride (mmol/L)	102 (96–111)	103 (96–113)
Aspartate aminotransferase (IJ/L)	50 (19–72)	46 (22–71)
Alanine aminostransferase (IJ/L)	30 (10–140)	30 (9–52)
**TYPE OF HOSPITALIZATION**		
Hospitalization^*^	14 (21.5%)	19 (29.2%)
Day hospital^**^	45 (69.2%)	38 (58.5%)
Day hospital and hospitalization^***^	6 (9.2%)	8 (12.3%)

* *Hospitalization means that the child stayed in the hospital continuously for longer than 24 h*.

** *Day hospital means that the child was admitted to the hospital, stayed in it for <24 h, and could be checked and treated in the hospital in the next few days, if required*.

*** *Day hospital and hospitalization means that the child could be moved from day hospital to full admission if the condition deteriorates or parents were unable to come for checks. In converse, a child who was initially hospitalized could be moved to day hospital once condition improved*.

**Figure 2:**
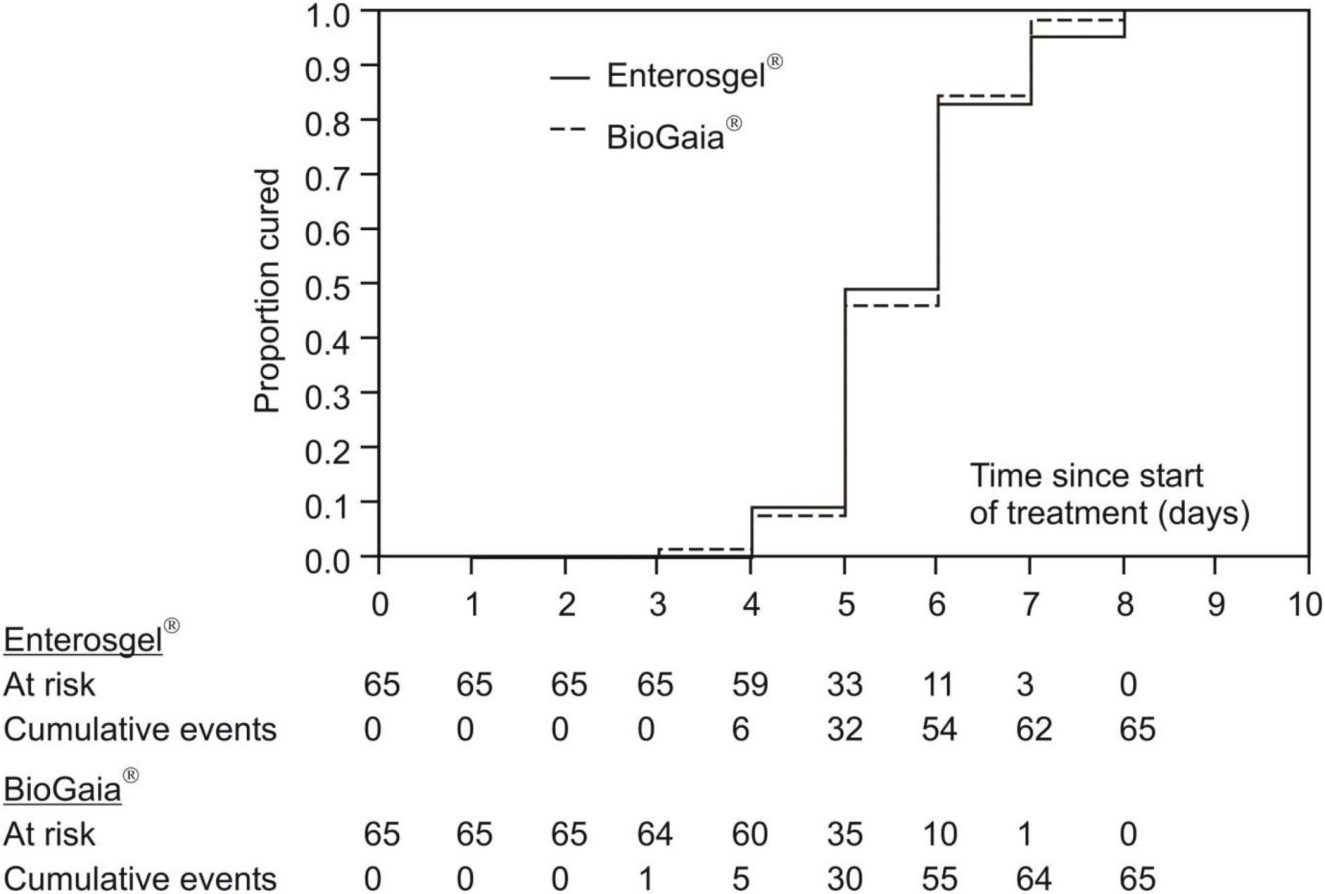
Kaplan–Meier curves of cumulative recovery in the PMSPH (Enterosgel®) and *L. reuteri* (BioGaia®) treatment groups for children with RVGE.

The primary measure of efficacy was the time in days till recovery. Figure 2 summarizes cumulative achievement of recovery in the two therapeutic groups: all the children recovered at the latest by the eighth day since the onset of the illness with comparable dynamics in both groups. Kaplan–Meier estimation of the median of the time of recovery was 6 days in both groups.

Table 2 shows results of the univariate and multivariate comparison of the two treatments (treatment 1: *L. reuteri*; treatment 2: PMSPH) according to the (current) risk of recovering from the rotavirus gastroenteritis (results of proportional hazards regression are condensed). Without any adjustment for any type of covariates (univariate model), current risk (probability) of recovery with PMSPH was not significantly different to that with *L. reuteri* (HR = 0.94, 95% CI 0.65–1.36; *p* = 0.754). Comparable results were achieved for the two multivariate models—Model 1 in Table 2 (HR = 0.89, 95% CI 0.60–1.32; *p* = 0.567) where, by the other covariates, Vesikari score was included and Model 2 in Table 2 (HR = 1.06; 95% CI 0.75–1.49; *p* = 0.816) where, by the other covariates, Clark's score was included (instead of Vesikari score).

**Table 2:** The results of univariate and multivariate analysis of the two treatments according to the (current) risk of recovery from rotavirus gastroenteritis.

	**HR (95% CI)**	***P***
**Univariate model**		
PMSPH vs. *L. reuteri*	0.94 (0.65–1.36)	0.754
**Multivariate model 1**		
PMSPH vs. *L. reuteri*	0.89 (0.60–1.32)	0.567
Age (for 2 months)^*^	1.11 (1.05–1.16)	<0.001
Boys (vs. girls)	0.68 (0.45–1.03)	0.069
Hospital infection (vs. community-acquired infection)	0.07 (0.03–0.16)	<0.001
Gastrointestinal co-infection (vs. no co-infection)	0.49 (0.15–1.63)	0.242
Day of illness at the beginning of treatment (for 1 day)	1.30 (0.95–1.78)	0.102
Vesikari score (for two points)^**^	0.49 (0.38–0.63)	<0.001
**Multivariate model 2**		
PMSPH vs. *L. reuteri*	1.06 (0.75–1.49)	0.816
Age (for 2 months)	1.07 (1.03–1.12)	0.002
Boys (vs. girls)	0.71 (0.48–1.04)	0.078
Hospital infection (vs. community-acquired infection)	0.05 (0.02–0.11)	<0.001
Gastrointestinal co-infection (vs. no co-infection)	0.83 (0.19–2.51)	0.834
Day of illness at the beginning of treatment (for 1 day)	1.42 (1.02–1.95)	0.036
Clark score (for two points)^***^	0.65 (0.54–0.79)	<0.001

* *For every 2 months, the chance of recovery was higher by 11%*.

** *For every two points, the chance of recovery was reduced by 51%*.

*** *For every two points, the chance of recovery was reduced by 35%*.

**Figure 3:**
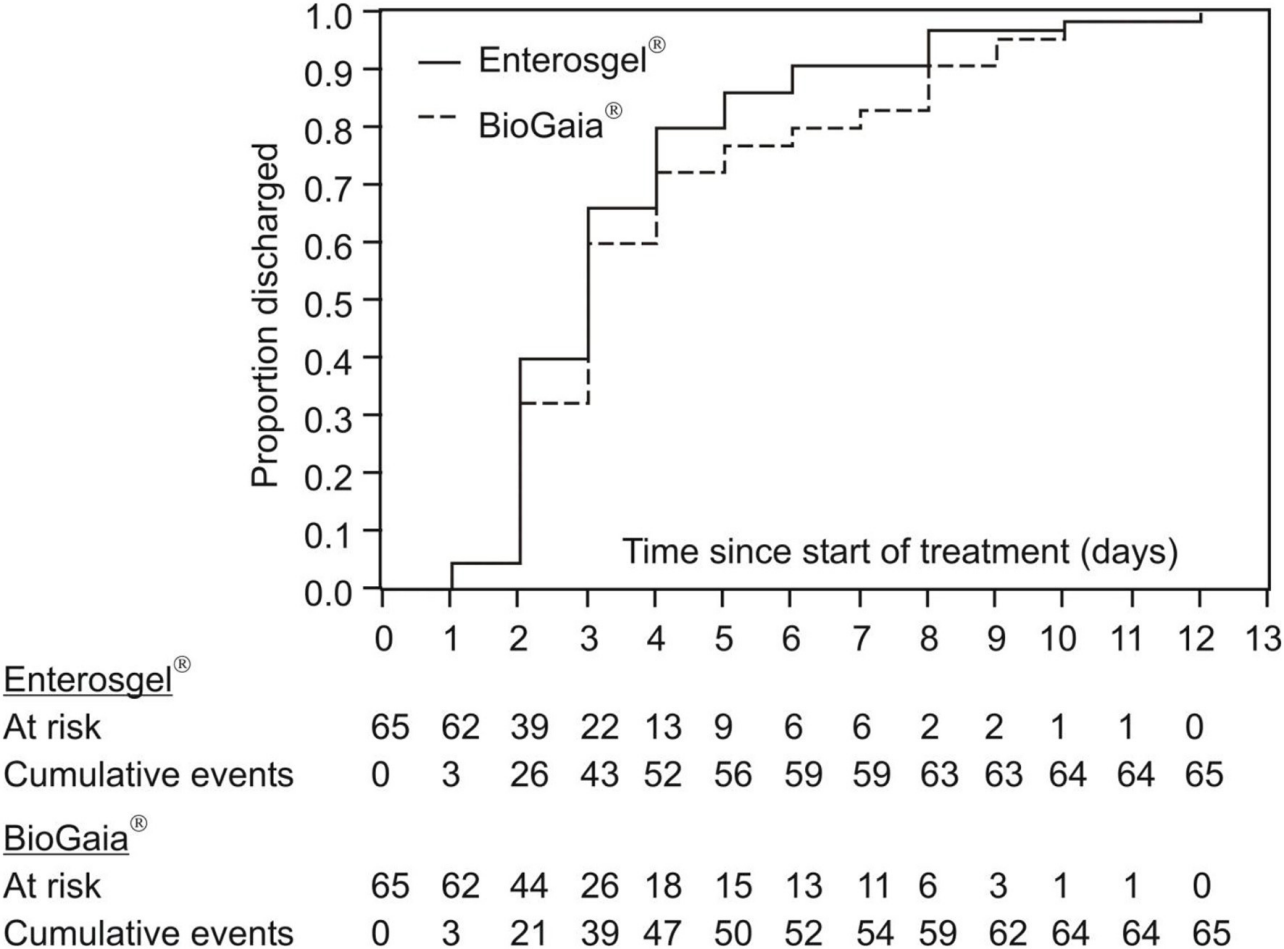
Kaplan–Meier curves of cumulative hospital discharge for the PMSPH (Enterosgel®) and *L. reuteri* (BioGaia®) treatment groups for children with RVGE.

The secondary measure of efficacy of the two treatments was the time from the start of study treatment to hospital discharge. Figure 3 summarizes the cumulative achievement of the cessation of hospital stay: all children were discharged by day 12 from the start of treatment, with dynamics completely comparable for the two treatments. The Kaplan–Meier estimation of the median time to hospital discharge was 3 days for both groups.

Table 3 summarizes the results of two multivariate models of proportional hazards regression, with identical variables to those in Table 2, except in this table, the time to discharge from hospital was analyzed. In both models, the first that includes the Vesikari score and the second that includes the Clark score, there was no significant difference between the two treatments according to the (current) risk of discharge from hospital: HR = 1.17 (95% CI 0.80–1.72; *p* = 0.419) and HR = 1.25 (95% CI 0.86–1.83; *p* = 0.239).

**Table 3:** The results of the multivariate comparison of the two treatments according to the (current) risk of hospital discharge of children with RVGE.

	**HR (95 % CI)**	***P***
**Multivariate model 1**		
PMSPH vs. *L. reuteri*	1.17 (0.80–1.72)	0.419
Age (for 2 months)	1.07 (1.02–1.11)	0.005
Boys (vs. girls)	0.67 (0.46–0.99)	0.041
Hospital infection (vs. community-acquired infection)	0.07 (0.03–0.15)	<0.001
Gastrointestinal co-infection (vs. no co-infection)	0.49 (0.11–1.40)	0.242
Day of illness at the beginning of treatment (for 1 day)	1.30 (0.95–1.79)	0.102
Vesikari score (for two points)^*^	0.82 (0.74–0.92)	<0.001
**Multivariate model 2**		
PMSPH vs. *L. reuteri*	1.25 (0.86–1.83)	0.239
Age (for 2 months)	1.07 (1.03–1.12)	0.002
Boys (vs. girls)	0.71 (0.48–1.04)	0.078
Hospital infection (vs. community-acquired infection)	0.05 (0.02–0.11)	<0.001
Gastrointestinal co-infection (vs. no co-infection)	0.83 (0.19–2.51)	0.834
Day of illness at the beginning of treatment (for 1 day)	1.42 (1.02–1.95)	0.036
Clark score (for two points)^**^	0.81 (0.74–0.89)	<0.001

* *For every two points, the chance for discharge from hospital was reduced by 18%*.

** *For every two points, the chance for discharge from hospital was reduced by 19%*.

Altogether, the statistical analyses of the length of hospital stay follow those of the primary outcome measure of treatment efficacy, which means no difference between the two treatments. Both analyses gave additional insights: the chances of recovery and hospital discharge were higher in older children and lower in the case of hospital-acquired RV infection, whereas the chances of both were lower with more severe disease according to the Vesikari score or Clark score for every two points gained. Model 2 also shows that chance of discharge from hospital was higher if the treatment was commenced later (42% relatively), possibly reflecting the partially spontaneous nature of recovery from the course of the illness.

The third measure of efficacy was the total number of stools during the illness (from the first symptom of RVGE till the end of survey, i.e., 8 days). In the group of children treated with PMSPH, the median number of stools was 23 (4–86), and in the group treated with *L. reuteri*, this was 25 (6–86); there was no significant difference between the treatment groups (95% CI −6 to 3), *p* = 0.479 (Mann–Whitney test).

The total number of adverse events (AEs) reported was one in the PMSPH group and none in the *L. reuteri* group. In the PMSPH group, one case of transient urticaria was reported, which coincided with the first dose of treatment. The urticaria was mild and self-limiting and did not recur with the second dose, and there was no need for cessation of treatment. This was likely not related to the intervention but to the condition itself. Three children refused to take PMSPH, which resulted in their exclusion from the study. This may have been the result of PMSPH palatability or the relatively larger volume of the treatment (20–30 ml) compared to only 20 drops (ca. 1 ml) of *L. reuteri*.

## Discussion

Due to the lack of universal RV vaccination in the national immunization program, RVGE is still one the most common reasons for pediatric emergency department visits in Croatia. The mainstay of treatment of RVGE is ORT. However, children often refuse to drink ORT, which leads to prostration and serious concern of parents/caregivers. In that case, the solution can be short-term intravenous parenteral rehydration. Worried parents often ask for some kind of adjunct therapy to shorten the duration of illness. In our study, we compared two such adjunct therapies for the treatment of RVGE.

### Principal Findings

This is the first study comparing possible therapeutic impact of the novel enterosorbent (PMSPH) with the recommended (probiotic) treatment of RVGE in children. The study showed that for the duration of illness, the length of hospital stay, and the total number of liquid stools, the results did not differ between the PMSPH and probiotic groups during the testing period. The study also confirmed good safety profile of the PMSPH, as demonstrated by the number of AE—only one case of mild urticaria (most likely related to the underlying condition).

### The Idea of the Study and the Comparison With Other Studies

When the new medical device PMSPH entered the Croatian market (2012), the idea to compare it with the probiotic treatment for RV was conceived. Compared with activated charcoal, PMSPH is a more potent absorbent in its binding ability toward high-molecular-weight compounds such as proteins and bacterial endotoxins (13, 23).

*L. reuteri* was chosen as a comparator because it was the first approved probiotic for the treatment of diarrhea in infants. *L. reuteri* was given in triple recommended dose because some studies showed a correlation between larger doses and a shorter duration of diarrhea, and we intended to maximize the probability of treatment efficacy (24).

Children aged 6–36 months were selected as it was most likely to be their first RV infection and consequently a more severe course of the disease. Children vaccinated against RV were excluded along with previous history of RV illness and children with chronic comorbidities or immune deficiencies, to eliminate any condition that could impact the disease course.

The children in both groups were given tested adjunct therapies alongside ORT or, initially, alongside intravenous rehydration fluids.

Our study did not show the advantage of either treatments as there was no significant difference in the duration of symptoms, with the median duration of symptoms till recovery at 6 days (HR = 0.94, 95% CI 0.65–1.36; *p* = 0.754). Search of the literature shows quite wide variations in the reported duration of RV illness without any intervention. In the study by Uhnoo et al. (25), the symptoms of RVGE in children lasted for 5.9 days. A second study claimed the duration of symptoms of 4–8 days (26), while a third found that symptoms could last from 2 to 22 days (27). Considering these conflicting data, it is difficult to ascertain if there is any additional benefit to either intervention, if rotavirus illness can last for 5.9 days. However, a Cochrane analysis of 63 trials analyzing probiotic efficacy in the treatment of acute infectious diarrhea (of these, 56 trials recruited infants and young children) showed a mean shortening of diarrhea of 25 h and one stool less on day 2 from starting therapy. Of course, results vary between studies depending on the subject age (children vs. adults), the country where the study took place (developed country vs. developing country), type of probiotic/s, or the type of causative agent of diarrhea (11).

Many studies have shown beneficial effects of probiotics on the course of acute infectious diarrhea, shortening duration of symptoms, reducing the number of liquid stools, and reducing hospital stay (5–9). Data relating to probiotic treatment efficacy are quite varied. Canani et al. compared five best-selling probiotics in Italy, with ~100 participants per group, who received either placebo (simple oral rehydration solution) or a probiotic. The study showed that only probiotics that contained LGG or a mix of four strains (*L. delbrueckii* var. *bulgaricus, L. acidophilus, S. thermophilus*, and *B. bifidum*) obtained a statistically significant benefit, resulting in shortening of symptom duration and reducing daily stool output. Interestingly, the *S. boulardii* probiotic recommended by ESPGHAN/ESPID did not obtain an advantage over placebo (20). Other studies did not show any beneficial effects of LGG (28, 29) or the drug racecadotril (30), also suggested by ESPGHAN/ESPID to be considered in the treatment of childhood diarrhea. Recent investigation of the treatment efficacy of *L. reuteri* in AGE showed failure to shorten symptoms duration, but had some effectiveness in the reduction of hospital stay (31). Taking into account many recent studies, the ESPGHAN workgroup has published new clinical guidelines for using probiotics in the treatment of AGE. The recommended probiotics remain the same (with the exception of *L. acidophilus* LB, which is replaced with a combination of *L. rhamnosus* 19070-2 and *L. reuteri* DSM 12246). However, the recommendation grade determined from the current evidence changed from strong to weak for all four recommended probiotics (32). Considering the similar, relatively high price of both *L. reuteri* and PMSPH, which is about 20 euros, and only minor benefit, we can question the reason for the recommendation of these adjunct therapies in low-income countries.

The second measure of efficacy was the duration in days spent in the hospital/day hospital visits. This parameter provides further evidence about possible influence of the treatment on the course of illness, although it is not a direct measure. Some children can have *a longer* hospital stay than RVGE symptoms (for example, children who were hospitalized for other reasons), or *shorter* (for example, children with benign course of the illness where brief hospitalization is sufficient). The length of hospital stay can be indicative of the entire disease course and the potential impact of the treatment. Rosenfeldt et al. showed a positive effect of probiotics (*L. reuteri* + LGG) not only on the duration of symptoms but also on the shortening of hospital stay by 48% (33). They used preparations with a higher probiotic concentration (10^10^–10^11^ CFU/day) than this study. However, we were not able to show a significant difference in the hospital stay between the two treatment groups (HR = 1.17 95% CI 0.80–1.72; *p* = 0.419 with Vesikari score included and HR = 1.25 95% CI 0.86–1.83; *p* = 0.239 with Clark score included).

The third measure of efficacy was the total number of liquid stools during the illness. As some adjunct therapies for infectious AGE, such as the antisecretory drug racecadotril, have an effect on reducing stool output, we also analyzed this parameter. This study found no significant difference in the number of stools between the two treatment groups (95% CI −6 to 3, *p* = 0.479; Mann–Whitney test).

The study did not record any AEs of the two treatments, except mild urticaria in one patient, which coincided with the first dose of PMSPH. We took it into account albeit this was unlikely to be the possible side effect of PMSPH, having in mind that this could be the consequence of RV infection itself.

## Strengths and Limitations

This study had a few limitations. The main limitation is the small sample size. Because of this, the safety profile of the new drug should be taken with some reservations. The second limitation is the study design itself, which was not a double-blind placebo-controlled study but an open-label, randomized, controlled study. We could not conduct a blinded treatment because of the different physical characteristics of the treatments (drops vs. gel). The same applied to the absence of a placebo group. It would have been unethical to have an intervention placebo control group and would have hindered recruitment to the study as it would be difficult to persuade parents (or caregivers) to consent to the study, knowing that their child could be given no treatment.

Another limitation was the method of measurement of diarrhea symptoms as the number of liquid stools rather than mass or volume, which is a more proportional measure of water loss. This measure was recorded by parents, and measuring volume or mass of stool would have required obligatory (unnecessary) hospitalization for 7–8 days, which was viewed as unethical.

On the positive side, the study population was very homogeneous as all subjects had similar clinical characteristics, thus making the comparison of the treatment efficacy more reliable.

## Conclusion

This randomized, open-label, clinical controlled trial showed no significant difference in the therapeutic efficacy between the new enterosorbent PMSPH and probiotic *L. reuteri* in the treatment of RVGE in children. The two treatments have similar excellent safety profiles in young aged children. As probiotics have some contraindications (congenital or acquired immune deficiencies, heart valve defects, and damaged bowel mucous membranes), PMSPH could be used in these instances as a safe and effective alternative to probiotics. In summary, the results of our study support the use of PMSPH as *adjunct* therapy alongside ORT in the management of RVGE in children aged 6 months and older. However, further investigations are recommended to define treatment efficacy in comparison to different treatment options and populations.

## Data Availability Statement

The original contributions presented in the study are included in the article/supplementary materials, further inquiries can be directed to the corresponding author/s.

## Ethics Statement

The studies involving human participants were reviewed and approved by the ethical committee of the University Hospital for Infectious Diseases, Zagreb, Croatia. Written informed consent to participate in this study was provided by the participants' legal guardian/next of kin.

## Author Contributions

LM and GT designed the study and participated in examinees' recruitment. IK and LS participated in examinees' recruitment. TK designed the database. VT made statistical analysis and interpreted the patient data. All authors contributed to the article and approved the submitted version.

## Conflict of Interest

The authors declare that the research was conducted in the absence of any commercial or financial relationships that could be construed as a potential conflict of interest.

## Abbreviations

AE: adverse events
AGE: acute gastroenteritis
CFU: colony-forming units
ESPGHAN: European Society for Pediatric Gastroenterology, Hepatology and Nutrition
ESPID: European Society for Pediatric Infectious Diseases
ITT: intention-to-treat
LGG: *Lactobacillus rhamnosus* GG
NSP4: nonstructural protein 4
ORT: oral rehydration therapy
PMSPH: polymethylsiloxane polyhydrate
RV: rotavirus
RVGE: rotavirus gastroenteritis
UHID: University Hospital for Infectious Diseases.
